# Comparative genome analysis of *Pediococcus damnosus* LMG 28219, a strain well-adapted to the beer environment

**DOI:** 10.1186/s12864-015-1438-z

**Published:** 2015-04-03

**Authors:** Isabel Snauwaert, Pieter Stragier, Luc De Vuyst, Peter Vandamme

**Affiliations:** Laboratory of Microbiology, Ghent University, K.L. Ledeganckstraat 35, B-9000 Ghent, Belgium; Research Group of Industrial Microbiology and Food Biotechnology (IMDO), Department of Bioengineering Sciences, Vrije Universiteit Brussel, Pleinlaan 2, B-1050 Brussels, Belgium

**Keywords:** *Pediococcus damnosus*, Illumina sequencing, Beer environment, Draft genome, Comparative genomics

## Abstract

**Background:**

*Pediococcus damnosus* LMG 28219 is a lactic acid bacterium dominating the maturation phase of Flemish acid beer productions. It proved to be capable of growing in beer, thereby resisting this environment, which is unfavorable for microbial growth. The molecular mechanisms underlying its metabolic capabilities and niche adaptations were unknown up to now. In the present study, whole-genome sequencing and comparative genome analysis were used to investigate this strain’s mechanisms to reside in the beer niche, with special focus on not only stress and hop resistances but also folate biosynthesis and exopolysaccharide (EPS) production.

**Results:**

The draft genome sequence of *P. damnosus* LMG 28219 harbored 183 contigs, including an intact prophage region and several coding sequences involved in plasmid replication. The annotation of 2178 coding sequences revealed the presence of many transporters and transcriptional regulators and several genes involved in oxidative stress response, hop resistance, *de novo* folate biosynthesis, and EPS production. Comparative genome analysis of *P. damnosus* LMG 28219 with *Pediococcus claussenii* ATCC BAA-344^T^ (beer origin) and *Pediococcus pentosaceus* ATCC 25745 (plant origin) revealed that various hop resistance genes and genes involved in *de novo* folate biosynthesis were unique to the strains isolated from beer. This contrasted with the genes related to osmotic stress responses, which were shared between the strains compared. Furthermore, transcriptional regulators were enriched in the genomes of bacteria capable of growth in beer, suggesting that those cause rapid up- or down-regulation of gene expression.

**Conclusions:**

Genome sequence analysis of *P. damnosus* LMG 28219 provided insights into the underlying mechanisms of its adaptation to the beer niche. The results presented will enable analysis of the transcriptome and proteome of *P. damnosus* LMG 28219, which will result in additional knowledge on its metabolic activities.

**Electronic supplementary material:**

The online version of this article (doi:10.1186/s12864-015-1438-z) contains supplementary material, which is available to authorized users.

## Background

Beer is a fermented beverage that is high in ethanol, carbon dioxide and flavorful yeast metabolites, contains hop-derived flavor and antimicrobial compounds, and is low in pH, oxygen, and residual nutrients [1]. This environment has selected for unique groups of bacteria specialized in growth in beer, including several species of lactic acid bacteria (LAB). Overall, one of the key metabolic actions of LAB is the reduction of pyruvate into lactate to regenerate NAD^+^ by means of lactate dehydrogenase activity. Depending on the beer type produced, lactate production may or may not be desired [2-5]. As most LAB species tolerate high ethanol concentrations and a low pH, hop resistance is the major factor limiting their growth in beer [1]. Hop-derived antimicrobial compounds, known as iso-alpha acids, cause permeability changes in the bacterial cell wall [6], leakage of the cytoplasmic membrane and subsequent inhibition of respiration and protein, DNA, and RNA syntheses [7], as well as changes in leucine uptake and proton ionophore activity [8]. Additionally, iso-alpha acids alter the redox properties of the bacterial cell, causing oxidative stress [9]. Accordingly, hop resistance is a multifactorial property that requires different mechanisms to counteract the action of iso-alpha acids. A well-known factor mediating hop resistance is the ATP-binding cassette multidrug transporter, HorA, which extrudes iso-alpha acids [10] and is plasmid-encoded [11].

LAB species frequently encountered in the beer environment belong to the genera *Lactobacillus* and *Pediococcus*, which are both Gram-positive, facultative anaerobic, chemo-organotrophic bacteria of the family *Lactobacillaceae* of the *Firmicutes* phylum. The species with the highest capacity to grow in beer are *Lactobacillus brevis* and *Pediococcus damnosus* [1], although the ability of bacteria to grow in beer is a strain- rather than species-specific characteristic [12]. Recent studies have investigated the mechanisms of *L. brevis* for overcoming stresses in beer by means of reverse transcription quantitative polymerase chain reaction (RT-qPCR) and proteomic analyses [13,14]. Less is known about the adaptations of *P. damnosus* to the beer environment. So far, *Pediococcus claussenii* ATCC BAA-344^T^ originating from spoiled beer, *Pediococcus pentosaceus* ATCC 25745 of plant origin, and *P. pentosaceus* IE-3 originating from a dairy effluent sample, are the only members of the genus *Pediococcus* for which the genome has been sequenced completely [15-17]. However, draft genome sequences are available for several strains of *Pediococcus acidilactici*, namely strain MA 18/5 M originating from animal food, strain DSM 20284 isolated from barley, strain 7_4 from a human fecal sample, and strain NGRI 0510Q, formerly known as *Pediococcus lolii* [18], isolated from ryegrass silage (http://genomesonline.org). In general, *Pediococcus* species possess rather small genomes (approximately 2 Mb), encoding a broad repertoire of transporters for efficient carbon and nitrogen acquisition and reflecting a limited range of biosynthetic capabilities. This suggests both extensive gene loss as well as acquisitions via horizontal gene transfer during the evolution of pediococci within their habitats [17]. Several strains possess plasmids containing genes regulating the fermentation of carbohydrates and encoding different types of resistances (*e*. g., resistance to stress, hop, or antibiotics) [17,19].

The strain *P. damnosus* LMG 28219 was isolated in 2013 from a Flemish acid beer, i.e. a sour (containing both lactic acid and acetic acid) and ethanolic [5-6% (vol/vol)] beer at the end of its maturation phase. A microbial diversity analysis of this mature beer proved that this strain was present in high numbers and is therefore able to replicate in this environment (unpublished results). Flemish acid beers are produced by a mixed-culture fermentation and represent culturally important products, for which microbial activities play critical roles in beer production and quality formation [3,5]. In the present study, the draft genome sequence of *P. damnosus* LMG 28219 is presented and analysed to obtain insights into its genome-based metabolic features. A better understanding of the molecular mechanisms underlying its metabolic capabilities enabled detailed insights into the mechanisms of adaptation of this strain to the beer environment. Furthermore, comparison of *P. damnosus* LMG 28219 with other sequenced genomes of members of the genus *Pediococcus* addressed the potentially unique properties of this strain and other strains adapted to the beer environment.

## Results and discussion

### General architecture and annotation of the *Pediococcus damnosus* LMG 28219 draft genome

Paired-end sequencing of the *P. damnosus* LMG 28219 genomic DNA yielded 3,137,316 reads that were assembled into 183 contigs [N50 of 24,659 base pairs (bp)], consisting of 69 large (>10,000 nucleotides) and 114 small (<10,000 nucleotides) contigs. This genome hence represents an intermediate size among the LAB [15,17]. An overview of the GC content and the length of the contigs is presented in Additional file 1. The GC content of the complete draft genome averaged 38.2 mol%. A total of 91 contigs could be mapped onto the *P. claussenii* ATCC BAA-344^T^ chromosomal DNA and were ordered accordingly (Table 1). Two clustered regularly interspaced short palindromic repeat (CRISPR) arrays were found on contigs 71 and 150, whereas three CRISPR-associated coding sequences (CDSs) (AH70_09625, AH70_09630, and AH70_09635) were found on contig 71, indicating that strain LMG 28219 acquired resistance to a plasmid or phage infection. One intact prophage region (GC content, 39.3%; region length, 39.4 kb) containing 50 CDSs was predicted and identified as the *Lactobacillus* phage Sha1, which was originally isolated from kimchi [20]. This phage has an isomeric head and a long tail and is classified as a member of the large family of *Siphoviridae*. The genes of phage Sha1 are organized into five functional clusters: replication/regulation/modification, packaging, structure/morphogenesis, lysis, and lysogeny. Most of the phage-related CDSs were found on contig 7, which was predicted to be part of the chromosomal DNA.

**Table 1:** **Overview of the** ***Pediococcus damnosus*** **LMG 28219 draft genome and the best BLAST hits**

**Draft genome of** ***P. damnosus*** **LMG 28219**	**Mean GC content (%)**	**Mean size consensus (bp)**	**Best BLAST hit**	
82, 98, 145, 116, 77, 119, 47, 53, 5, 89, 56, 76, 166, 84, 81, 136, 102, 141, 72, 30, 52, 129, 74, 54, 19, 110, 147, 49, 26, 85, 137, 61, 146, 177, 7, 68, 131, 104, 87, 32, 17, 80, 66, 165, 2, 20, 101, 96, 4, 59, 45, 164, 118, 64, 176, 69, 90, 24, 127, 10, 46, 139, 114, 60, 124, 23, 83, 51, 151, 169, 106, 11, 121, 78, 55, 43, 130, 48, 65, 168, 27, 62, 60, 79, 6, 152, 120, 73, 35, 99, 29, 71*	38.3	20814	chromosomal DNA (NC_016605)	***P. claussenii*** **ATCC BAA-344** ^**T**^
21, 39, 41, 183	36.9	4040	pPECL-3 (NC_016636)	
12, 21, 94, 95, 112, 135	38.5	3419	pPECL-4 (NC_016607)	
1,21, 39, 40, 95, 97, 107, 132, 135, 170	38.6	5198	pPECL-5 (NC_016608)	
21, 107, 135, 178	37.0	4924	pPECL-6 (NC_017017)	
**8**,21, 39, 41, 86, 94, **182**, 183	38.1	3926	pPECL-7 (NC_017018)	
**70**,**14**, 97*	42.5	9516	pPECL-8 (NC_017019)	
16, 57	49.0	2538	*Bacillus megaterium* QM B1551 (pBM400, NC_004604) and WSH-002 (WSH-002_p1, CP003018)	**Other**
123, 134, 140	37.2	4034	*L. brevis* 925A (pLB925A04, NC_012551), KB290 (pKB290-4, AP012171), and ATCC 367 (plasmid 1, NC_008498)	
37	44.6	1242	*L. plantarum* WCFS1 (pWCFS103, NC_006377)	
100, 122	33.9	5879	*L. buchneri* CD034 (pCD034-3, CP003044) and NRRL B-30929 (pLBUC01, NC_015420)	
18, 22, 93, 108, 109, 113, 115, 143, 158, 162, 163	40.6	2797	*Several Lactobacillus* spp.	

Contigs with the highest hit scores are highlighted in bold. *Contig numbers are mentioned in ordered fashion. p: plasmid, L.: *Lactobacillus*, P.: *Pediococcus*, T: type strain, bp: base pairs.

A BLAST search of the remaining non-chromosomally encoded contigs against a plasmid-specific database (*i.e.*, PATRIC) revealed many similarities towards plasmids of different bacterial species (see below). Many of these contigs (as listed in Table 1) were similar to the plasmids of *P. claussenii* ATCC BAA-344^T^, with the exception of plasmids pPECL-1 and pPECL-2, onto which no contigs mapped. These two plasmids are small and cryptic (1.85Kb and 2.45Kb in size, respectively), whereas the other six plasmids of *P. claussenii* ATCC BAA-344^T^ range from 16 to 36 kb and contribute to roughly 7% of the strain’s coding capacity [21]. Four, six, ten, four, eight, and three contigs mapped onto plasmids pPECL-3, pPECL-4, pPECL-5, pPECL-6, pPECL-7, and pPECL-8 of *P. claussenii* ATCC BAA-344^T^, respectively. A total of 19 contigs did not show high similarities towards the plasmids of *P. claussenii* ATCC BAA-344^T^. Contigs 16 and 57 were similar to plasmids pBM400 and WSH-002_p1 of *Bacillus megaterium* strains QM B1551 and WSH-002, respectively, whereas others were similar to plasmids of *L. brevis* strains 925A, KB290, and ATCC 367 (contigs 123, 134, and 140, respectively), *Lactobacillus plantarum* WCFS1 (contig 37), *Lactobacillus buchneri* strains CD034 and NRRL B-30929 (contigs 100 and 122, respectively), or other *Lactobacillus* species (all remaining contigs). In addition, several CDSs involved in plasmid replication were found, which encoded replication initiation proteins (AH70_10315 on contig 8, AH70_03600 on contig 21, AH70_00575 on contig 107, AH70_02015 on contig 138, and AH70_03140 on contig 182).

Gene finding and annotation of the *P. damnosus* LMG 28219 draft genome with the Integrated Microbial Genomics Expert review (IMG-RE) software resulted in a total gene count of 2266, among which 96.12% were CDSs. A total of 79.08% of the CDSs could be assigned to a protein, among which 23.39% and 12.80% were predicted to be enzymes and transporters, respectively. This agrees with the property of LAB to encode a broad repertoire of transporters for efficient nutrient uptake and reflects their limited biosynthetic capabilities [17]. Additionally, 2.60% of the genes encoded signal peptides, whereas 26.04% encoded transmembrane proteins. Furthermore, a total of 56 tRNA and three 5S, 16S, and 23S rRNA genes were predicted. As expected, most CDSs were involved in carbohydrate, protein, DNA, and RNA metabolism and in cell wall and capsule construction. These findings agreed with those of Makarova and colleagues [17], who analyzed 12 genomes belonging to the order *Lactobacillales* and defined a core of *Lactobacillales*-specific clusters of orthologous groups (LaCOGs). The functional distribution of the conserved core of 567 LaCOGs showed that the majority encodes components of the information processing systems (translation, transcription, and replication), which are likely to perform essential functions. With the exception of 9 LaCOGs (*i.e.*, COGs 0230, 0419, 0454, 0457, 0470, 0762, 2815, 2855, and 4608), all core LaCOGs were found in the draft genome of *P. damnosus* LMG 28219. Genome closure could possibly lead to the identification of these missing core functions, although an update of the core LaCOGs is mandatory, because only 12 *Lactobacillales* species out of more than 500 species of this order were included; in addition, only one of these 12 species belonged to the genus *Pediococcus*.

Next to these core functions, the draft genome sequence of *P. damnosus* LMG 28219 comprised 50 predicted CDSs that were associated with virulence, disease, and defense, including three multidrug resistance efflux pumps. Also, 42 CDSs related to stress response were detected, seven of which were involved in oxidative stress response. Four of the non-core LaCOGs present in the *P. damnosus* LMG 28219 draft genome comprised 12 or more CDSs in a COG, namely arabinose efflux permeases (COG2814), transcriptional regulators (COG1309), predicted transcriptional regulators (COG0789), and transposases and their inactivated derivatives (COG2826). Arabinose efflux permeases belong to the major facilitator superfamily, a large and diverse group of secondary transporters that includes uniporters, symporters, and antiporters [22]. A total of 12 and 10 CDSs of COG2814 were annotated as arabinose efflux permeases and drug resistance transporters from the EmrB/QacA subfamily, respectively. In addition, one CDS was predicted to be a drug resistance transporter of the Bcr/CflA subfamily and one was predicted to be a transporter belonging to the carbohydrate transporter family. The COG1309 members belonged to the TetR family of transcriptional repressors, which are involved in transcriptional control of multidrug efflux pumps, pathways for the biosynthesis of antibiotics, responses to osmotic stress and toxic chemicals, control of catabolic pathways, differentiation processes, and pathogenicity. Analysis of the CDSs of COG0789 did not reveal insights into their function. Finally, the CDSs in COG2826 were all transposases belonging to the insertion sequence *IS30* family. The presence of IS elements may lead to genomic instability due to its role in gene loss and gene gain [23].

### Comparative genomics

The draft genome sequence of *P. damnosus* LMG 28219 was compared with the complete genome sequences of *P. pentosaceus* ATCC 25745 and *P. claussenii* ATCC BAA-344^T^. The number of CDSs predicted in the draft genome of *P. damnosus* LMG 28219 was higher compared to those in the reference genomes (Table 2). In addition, the number of genes in *P. claussenii* ATCC BAA-344^T^ exceeded that of *P. pentosaceus* ATCC 25745. This could be explained by the need to acquire additional functions during evolution to be able to survive in the hostile beer environment compared to the plant environment. Possibly, *P. pentosaceus* ATCC 25745 can acquire more nutrients from its environment compared to *P. damnosus* LMG 28219 and *P. claussenii* ATCC BAA-344^T^. The number of rRNA operons in the genome of *P. damnosus* LMG 28219 was lower compared to that in *P. pentosaceus* ATCC 25745 and *P. claussenii* ATCC BAA-344^T^, which may reflect differences in ecological competitiveness [17,24,25]. In addition, the average nucleotide identities (ANIs) of *P. damnosus* LMG 28219 *versus P. pentosaceus* ATCC 25745 and *P. claussenii* ATCC BAA-344^T^ were 69.81% and 69.28%, respectively, whereas the ANIs between *P. pentosaceus* ATCC 25745 and *P. claussenii* ATCC BAA-344^T^ were 71.53%. These results agreed with previous findings, indicating that *P. claussenii* and *P. pentosaceus* are evolutionarily closer related to each other compared to *P. damnosus* [26].

**Table 2:** **Comparison of genome characteristics of** ***P. pentosaceus*** **ATCC 25745,** ***P. claussenii*** **ATCC BAA-344** ^**T**^ **, and** ***P. damnosus*** **LMG 28219 (based on the IMG-ER server)**

	***P. pentosaceus*** **ATCC 25745**	***P. claussenii*** **ATCC BAA-344** ^**T**^	***P. damnosus*** **LMG 28219**
**Accession number** *	**CP000422**	**CP003137** *	**JANK00000000**
Origin	Plant	beer	beer
Size	1,832,387	1,966,362	2,231,216
CDSs	1755	1892	2178
rRNA genes	5	4	3
tRNA genes	55	57	56
GC%	37.4	37.0	38.2

*Only the Genbank record of the chromosomal DNA is given, P.: *Pediococcus,* T: type strain, CDSs: coding sequences, IMG-ER: integrated microbial genomics expert review.

**Figure 1:**
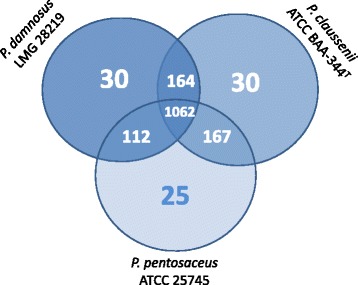
**Visualization of the OrthoMCL output comparing the number of unique and/or shared orthologs of** ***Pediococcus claussenii*** **ATCC BAA-344** ^**T**^ **,** ***P. pentosaceus*** **ATCC 25745, and** ***P. damnosus*** **LMG 28219. P.:** ***Pediococcus,*** **T: type strain.**

The predicted proteome of *P. damnosus* LMG 28219 was assigned into orthologous clusters, along with the proteomes of *P. claussenii* ATCC BAA-344^T^ and *P. pentosaceus* ATCC 25745 to predict unique and/or shared characteristics between these LAB strains and species. A total of 1062 putative orthologous proteins were shared between *P. pentosaceus* ATCC 25745 (of plant origin), *P. claussenii* ATCC BAA-344^T^ and *P. damnosus* LMG 28219 (both of beer origin), whereas 25, 30, and 30 orthologs were unique to *P. pentosaceus* ATCC 25745, *P. claussenii* ATCC BAA-344^T^, and *P. damnosus* LMG 28219, respectively (Figure 1; Additional file 2). Genes that are shared between *P. damnosus* LMG 28219 and *P. claussenii* ATCC BAA-344^T^, but not by *P. pentosaceus* ATCC 25745, possibly promote survival in beer. For instance, the presence of genes involved in hop resistance, osmotic stress response, exopolysaccharide (EPS) production, and the presence or absence of complete or partial metabolic pathways may lead to the strain’s ability to grow in beer. Furthermore, the functions enriched in the genomes of bacteria capable of growing in beer are likely to contribute to their specific phenotypic traits.

### Genes involved in hop resistance

**Figure 2:**
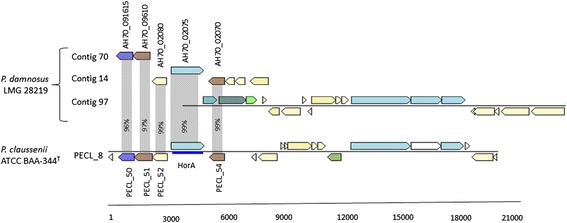
**Schematic comparative representation of plasmid pPECL-8 from** ***Pediococcus claussenii*** **ATCC BAA-344** ^**T**^ **and contigs 70, 14, and 90 of** ***P. damnosus*** **LMG 28219.** Each arrow indicates an open reading frame (ORF), the size of which is proportional to the length of the arrow. Coloring of the arrows represents different functions of the genes as indicated above each arrow. The amino acid identity of the relevant encoded proteins is indicated in percentages. The *horA* gene and its surrounding genes are indicated [from left to right: AH70_091615 (glycosyl transferase), AH70_09610 (glycosyl transferase), AH70_02080 (acyl-phoshate glycerol 3-phospate), HorA: AH70_02075 (multidrug transporter), and AH70_02070 (glycosyl transferase)]. P.: *Pediococcus*, T: type strain.

Research on hop resistance genes have suggested that these genes are typically acquired via horizontal gene transfer [27-29]. Although several putative hop resistance genes have been described, only the presence of *horA* definitively correlates with LAB growth in beer, with the presence of *horA* together with *hitA* and/or *horC* allowing fast bacterial growth. A recent transcriptome study of *P. claussenii* ATCC BAA-344^T^ showed increased transcript levels of several genes during growth in beer compared to growth in MRS-B medium [21]. This was especially the case for genes encoded on plasmids pPECL-3, pPECL-5, and pPECL-8. Contrasting to pPECL-3 and pPECL-5, many CDSs on pPECL-8 showed strong homology towards contigs 70, 14, and 97 in the draft genome of *P. damnosus* LMG 28219 (Figure 2). Plasmid pPECL-8 harbored the previously mentioned *horA* gene, which is involved in hop resistance. Furthermore, several orthologs of the *P. claussenii* ATCC BAA-344^T^*horA* gene were found both in the draft genome of *P. damnosus* LMG 28219 (AH70_02075 on contig 14 and AH70_01035 on contig 114) and in the complete genome of *P. pentosaceus* ATCC 25745 (YP_805121), which all encoded ABC-type multidrug transporters. In depth analysis of these CDSs revealed that only AH70_02075 showed high (99%) amino acid sequence homology towards the *horA* gene product of *P. claussenii* ATCC BAA-344^T^; the remaining CDSs showed less than 70% homology. Possibly, these *horA* orthologs are descendants of a common ancestor that evolved towards a different functionality, with the *horA* gene in *P. claussenii* ATCC BAA-344^T^ and AH70_02075 in *P. damnosus* LMG 28219 being specialized in the extrusion of iso-alpha acids.

Interestingly, several studies have revealed that the *horA* gene is consistently surrounded by the same set of genes in bacteria growing in beer, suggesting that these entire regions are acquired via horizontal gene transfer [21,28] and could possibly play a role in their adaptation to the beer niche. Most of these *horA*-surrounding genes are involved in phospholipid or cell wall biosynthesis. Orthologs of PECL_1950, PECL_1952, and PECL_1954 flanking the *horA* gene were found in the genome of *P. damnosus* LMG 28219 (AH70_09615 on contig 70 and AH70_02080 and AH70_02070 on contig 14, respectively) but not in the *P. pentosaceus* ATCC 25745 genome sequence. AH70_09615 and AH70_02080 were annotated as an acyl-phosphate glycerol 3-phospate and a glycosyl transferase, respectively. Both PECL_1950 and PECL_1952 are members of the lysophospholipid acyltransferase superfamily, which contains acyltransferases of *de novo* and remodeling pathways of glycerophospholipid biosynthesis. Finally, AH70_02070 contained a family 8 glycosyl transferase domain that catalyzes the transfer of sugar moieties from activated donor molecules to specific acceptor molecules, forming glycosidic bonds.

In addition to the *horA* gene, the *hitA* gene encodes a divalent cation transporter and confers hop resistance by importing manganese and thereby counteracting proton gradient dissipation [29]. Orthologs of the *hitA* gene of *L. brevis* L5784 (AB035808) were found in the genomes of *P. damnosus* LMG 28219 (AH70_05540), *P. claussenii* ATCC BAA-344^T^ (AEV94625.1), and *P. pentosaceus* ATCC 25745 (YP_805164). The reason for the presence of the *hitA* gene in *P. pentosaceus* ATCC 25745 of plant origin remains unclear. Furthermore, HorC has been suggested to be a proton motive force-dependent multidrug transporter, whose gene expression is under control of the HorB transcriptional regulator [30]. Orthologs of the *horC* gene of *Lactobacillus backii* LMG 23555 (BAF56899.1) were found on contig 18 (AH70_03090) in the *P. damnosus* LMG 28219 and *P. claussenii* ATCC BAA-344^T^ genome sequences (AEV95756.1), but not in that of *P. pentosaceus* ATCC 25745. Finally, *horB*, *bsrA*, and *bsrB* homologs were not found in the draft genome sequence of *P. damnosus* LMG 28219 [31].

### Folate biosynthesis

The beer isolates *P. damnosus* LMG 28219 and *P. claussenii* ATCC BAA-344^T^ contained a set of genes involved in folate biosynthesis that were absent in the genome of *P. pentosaceus* ATCC 25745 (plant origin). These included a 5,10-methylenetetrahydrofolate reductase (EC 1.5.1.20, AH70_04245 on contig 29), a dihydropteroate synthase (EC 2.5.1.15, AH70_05560 on contig 4), a 2-amino-4-hydroxy-6-hydroxymethyldihydropteridine pyrophosphokinase (EC 2.7.6.3, AH70_05580 on contig 4), a GTP cyclohydrolase I type 1 (EC 3.5.4.16, AH70_05575 on contig 4), and a dihydroneopterin aldolase (EC 4.1.2.25, AH70_05585 on contig 4).

**Figure 3:**
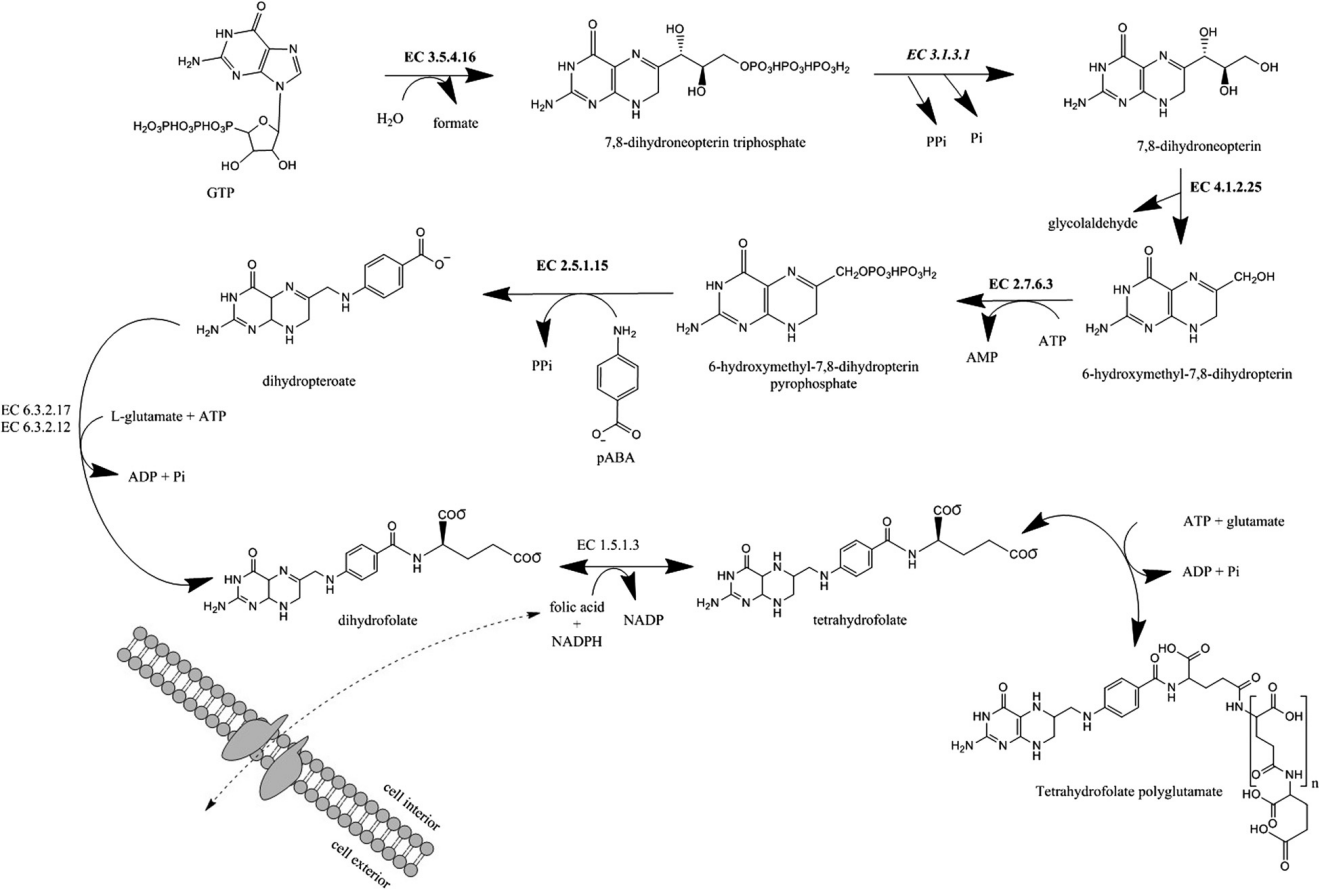
**Folate biosynthesis pathway reconstruction.** The enzymes found in both *Pediococcus damnosus* LMG 28219 and *Pediococcus claussenii* ATCC BAA-344^T^ are highlighted in bold, with the exception of EC 3.1.3.1 that was only found in the genome of *P. claussenii* ATCC BAA-344^T^ (highlighted in bold and italic). The remaining enzymes were shared by *P. damnosus* LMG 28219, *P. claussenii* ATCC BAA-344^T^, and *P. pentosaceus* ATCC 25745. The mechanism of the reaction highlighted with a dashed arrow is uncertain and needs further investigation. The figure was constructed using the ChemBioDraw software v. 13.0 (Perkin Elmer Inc., Waltham, MA, USA). P. *Pediococcus*, T: type strain, ATP: adenosine triphosphate, ADP: adenosine diphosphate, AMP: adenosine monophosphate, NADP(H): nicotinamide adenine dinucleotide phosphate, pABA: 4-aminobenzoic acid, P(P)i: (pyro)phosphate, EC: enzyme commission, GTP: guanoside triphospate.

Because folate-dependent formylation of the initiator tRNA is a hallmark of bacterial translation and because bacteria cannot import formylmethionyl-tRNA, folate is essential for bacterial growth [32]. Most bacteria make folate *de novo*, starting from GTP and chorismate [32]. The first enzyme in *de novo* folate biosynthesis is GTP cyclohydrolase I that catalyzes a complex reaction, in which the five-membered imidazole ring of GTP is opened and a six-membered dihydropyrazine ring is formed (Figure 3). The resulting 7,8-dihydroneopterin triphosphate is then converted into the corresponding monophosphate by a specific pyrophosphatase. A pyrophosphatase was found in the genome of *P. claussenii* ATCC BAA-344^T^ but was absent in the draft genome sequence of *P. damnosus* LMG 28219. BLASTp of the pyrophosphatase gene against the predicted CDS amino acid sequences of *P. damnosus* LMG 28219 did not reveal the presence of a homologous gene. Possibly, the gene performing this function in *P. damnosus* LMG 28219 has not been previously identified or is missing due to non-orthologous gene replacement. Alternatively, some missing information in the draft genome sequence of *P. damnosus* LMG 28219 may account for this gap in the folate biosynthesis pathway. Dihydroneopterin aldolase subsequently releases glycoaldehyde to produce 6-hydroxymethyl-7,8-dihydropterin, which is then pyrophosphorylated by hydroxymethyldihydropterin pyrophosphokinase. 6-Hydroxymethyl-7,8-dihydropterin pyrophosphate and 4-aminobenzoic acid moieties are condensed by dihydropteroate synthase and this results in the production of dihydropteroate. The enzymes involved in the conversion of dihydropteroate into tetrahydrofolate-polyglutamate are shared by the three genomes analyzed. These similarities and differences in folate biosynthesis potential between the *P. damnosus* LMG 28219*, P. claussenii* ATCC BAA-344^T^*,* and *P. pentosaceus* ATCC 25745 genomes may be explained by the environmental acquisition of folate by the latter strain. Plants produce folate *de novo*, so possibly *P. pentosaceus* ATCC 25745 harbors folate transporters to import folate produced by its host and lost genes involved in folate biosynthesis during evolution.

### Exopolysaccharide production

Laboratory experiments indicated that *P. damnosus* LMG 28219 produces EPS. The ability of a strain to produce EPS is not directly correlated to its ability to reside in beer but probably has importance in biofilm formation [12], thereby enabling persistence in the brewery environment. The EPS produced by *P. claussenii* ATCC BAA-344^T^ is a high-molecular-mass β-glucan produced by the action of a transmembrane glycosyl transferase (*gtf*) gene. This gene is located on plasmid pPECL-7, which is not essential for growth in beer [21]. The fibrillar polymer consists of a trisaccharide repeating unit with a β-1,3-linked glucose backbone and branches made up of single β-1,2-linked D-glucopyranosyl residues. Walling and colleagues [33] reported a glucosyl transferase gene (*dps*) in *P. damnosus* IOEB8801, originating from wine, that produces a linear backbone of 3-β-D-glucose-1 moieties. Surprisingly, no homologies towards the *gtf* and *dps* genes were found in the draft genome sequence of *P. damnosus* LMG 28219. Yet, contig 56 harbored a CDS (AH70_07835), containing a glycosyl transferase (group 2) domain, which may be involved in EPS production. OrthoMCL analysis indicated that this CDS was unique to *P. damnosus* LMG 28219. Other CDS candidates involved in EPS production by *P. damnosus* LMG 28219 were AH70_01405, AH70_01410, and AH70_01415, which were all predicted to be present on contig 122. These proteins did not show amino acid sequence homologies of more than 70% towards the proteomes of *P. claussenii* ATCC BAA-344^T^ and *P. pentosaceus* ATCC 25745. AH70_01405 is an EPS biosynthesis protein, consisting of an AAA domain containing a P-loop, and showed similarities towards the capsular EPS family protein (EHO53752) found in the genome of *Lactobacillus kisonensis* F0435, originating from the human oral cavity [34]. AH70_01410 is a lipopolysaccharide biosynthesis domain that is involved in the biosynthesis of EPS. This protein showed more than 70% protein sequence homology towards the chain length determinant protein (EEI18212) of *L. buchneri* ATCC 11577, isolated from the human oral cavity [35]. Finally, AH70_01415 harbored a cell envelope-related transcriptional attenuator domain, which describes a domain of unknown function that is found in the predicted extracellular domain of a number of putative membrane-bound proteins [36]. One of those is CpsA, a putative regulatory protein involved in EPS biosynthesis [36]. AH70_01415 showed more than 70% protein sequence homology towards a cell envelope-like function transcriptional attenuator common domain protein (EEI18211) of *L. buchneri* ATCC 11577 and a biofilm regulatory protein of *L. kisonensis* F0435 (see above). Besides the presence of proteins potentially involved in EPS production in *P. damnosus* LMG 28219 (as discussed above), the mechanism of EPS production in *P. damnosus* LMG 28219 remains unclear.

### Genes involved in oxidative stress response

Pittet and coworkers [21] found a set of highly transcribed genes in *P. claussenii* ATCC BAA-344^T^ during growth in beer as a response to the oxidative stress imposed by hops. These genes are manganese transport proteins, methionine sulfoxide reductases MsrA and MsrB as well as other metal transport and homeostasis proteins. Orthologs of these genes were found in the genomes of *P. damnosus* LMG 28219 and *P. pentosaceus* ATCC 25745, indicating that the presence of these genes is not unique to strains capable of growing in beer. These results indicate that growth in beer is a multifactorial reaction of the cell towards a challenging environment, not only involving the presence of specific genes but also the up-and down-regulation of specific sets of genes.

### COGs enriched in the genomes of bacteria originating from beer

Next to COGs unique to bacteria originating from beer, enriched COGs could also provide insight into the mechanisms of niche adaptation. The four most abundant non-core LaCOGs (COG 2814, COG1309, COG0789, and COG2826, discussed above) in the *P. damnosus* LMG 28219 draft genome were also present in the *P. claussenii* ATCC BAA-344^T^ and *P. pentosaceus* ATCC 25745 genomes, but were enriched only in the genomes of the bacterial strains originating from beer (*i.e.*, *P. damnosus* LMG 28219 and *P. claussenii* ATCC BAA-344^T^) (Table 3). Other enriched functions were COG1846 (transcriptional regulators), COG0596 (predicted hydrolases or acyltransferases of the alpha/beta hydrolase superfamily) and COG0745 (response regulators consisting of a CheY-like receiver domain and a winged-helix DNA-binding domain). The enrichment of the above-mentioned functions in bacteria growing in beer was further substantiated when analyzing additional genome sequences of the genus *Pediococcus* and *Lactobacillus*. Many COGs that were enriched in bacteria originating from beer are involved in transcriptional regulation, indicating that genes can be up- or down-regulated in hop-stressed cells. Currently, it is not clear in which processes these regulators are involved but this may be revealed by transcriptome analyses.

**Table 3:** **Overview of the functions enriched in the genomes of lactic acid bacteria (LAB) of beer origin compared to those of LAB not originating from beer (based on IMG-ER)**

		**No beer origin**							**Beer origin**			
**COG**	**Name**	***L. delbrueckii bulgaricus*** **ATCC 11842**	***P. acidilactici*** **7_4**	***P. acidilactici*** **DSM 20284**	***P. acidilactici*** **MA 18/5 M**	***P. acidilactici*** **NGRI 0510Q**	***P. pentosaceus*** **ATCC 25745**	***P. pentosaceus*** **IE-3**	***P. claussenii*** **ATCC BAA-344** ^**T**^	***P. damnosus*** **LMG 28219**	***L. malefermentans*** **KCTC 3548**	***L. rhamnosus*** **ATCC 8530**
COG2814	Arabinose efflux permease	8	18	19	18	19	16	16	22	24	23	26
COG1309	Transcriptional regulator	2	4	4	4	7	5	7	23	14	13	16
COG0789	Predicted transcriptional regulator	2	6	5	6	5	5	4	8	12	9	6
COG2826	Transposase and inactivated derivatives, *IS30* family	4	0	1	1	1	1	2	4	12	10	4
COG1846	Transcriptional regulator	1	5	5	5	6	6	7	10	11	16	9
COG0596	Predicted hydrolases or acyltransferases (alpha/beta hydrolase superfamily)	3	2	2	2	3	2	2	4	8	4	8
COG0745	Response regulators consisting of a CheY-like receiver domain and a winged-helix DNA-binding domain	5	5	5	5	5	5	5	7	8	6	12

The number of CDSs in the different COGs are listed in the table. T: type strain, P. *Pediococcus*, L. *Lactobacillus,* CDS: coding sequence, IS: insertion sequence.

## Conclusions

The draft genome of *P. damnosus* LMG 28219 and its comparative analysis with the genomes of other pediococci provided insights into the adaptation of this strain to the beer environment. These adaptations included the presence of the *horA* gene and its surrounding genes, genes involved in *de novo* folate biosynthesis, genes involved in the production of EPS, and the enrichment of functions related to transcriptional regulation. Furthermore, the results presented in this study will enable future transcriptome analysis of *P. damnosus* LMG 28219, which can provide additional insights into its metabolic activities.

## Methods

### Bacterial strain and growth conditions

A mature Belgian red-brown acidic ale brew sample was serially diluted in 0.86% (wt/vol) saline and 50 μL of each dilution was plated on de Man-Rogosa-Sharpe (MRS, Oxoid, Basingstoke, Hampshire, United Kingdom) agar medium, supplemented with 5 ppm amphotericin B (Sigma-Aldrich) and 200 ppm cycloheximide (Sigma-Aldrich) to favor the growth of LAB and inhibit yeast growth. Agar plates were incubated at 28°C in an aerobic atmosphere for 4–6 days. LAB isolates were identified through sequence analysis of the 16S rRNA and phenylalanyl-tRNA synthase alpha subunit (*pheS*) genes [26]. Strain *P. damnosus* LMG 28219 was present in large numbers in the mature brew sample (data not shown) and was used for genome sequencing. To obtain cell pellets, *P. damnosus* LMG 28219 was propagated in 750 ml de Man-Rogosa-Sharpe broth (MRS-B; Oxoid) at 28°C for 3 days, followed by microcentrifugation (11000 rpm, 15 min, 4°C).

### DNA extraction and Illumina sequencing

Total DNA was extracted using a procedure applied by Gevers and colleagues [37], with several modifications. (i) The thawed pellet was washed in 1 ml TES buffer (6.7% sucrose, 50 mM Tris–HCl, pH 8.0, 1 mM EDTA) and resuspended in 275 μl STET buffer (8.0% sucrose, 5.0% Triton X-100, 50 mM Tris–HCl, pH 8.0, 50 mM EDTA). (ii) Ninety μl lysozyme-mutanolysin-proteinase K solution [TES buffer containing 1667 U/ml mutanolysine (Sigma-Aldrich, St. Louis, MO, USA), 33 mg/ml lysozyme (Serva, Heidelberg, Germany), and 2.78 mg/ml proteinase K (Merck, Darmstadt, Germany)] was added and the suspension was incubated at 37°C for 1 h. (iii) Prior to extraction with a phenol/chloroform/isoamylalcohol (49.5:49.5:1.0) solution, proteins were precipitated by adding 250 μl ammonium acetate. (iv) Five μl RNase (2 mg/l, Sigma-Aldrich) was added to the DNA solution, which was incubated at 37°C for 60 min and stored at −20°C. The integrity, concentration, and purity of the DNA isolated was evaluated using 1.0% (wt/vol) agarose gels, stained in ethidium bromide, and by spectrophotometric measurements at 234, 260, and 280 nm. A fluorescent stain-based kit (Qubit® dsDNA Broad range assay kit; Life Technologies, Carlsbad, CA, USA) was used for an accurate determination of the DNA concentration. Because of the low DNA concentration obtained, the extraction procedure was performed in triplicate and the resulting DNAs were pooled, after which an additional purification [by adding 12.5 μl sodium acetate (3.0 M, pH 4.8) and 200 μl 95% ice-cold ethanol to 100 μl DNA product] and quantification step was performed.

### Illumina sequencing and genome assembly

Library preparation and genome sequencing was performed by BaseClear BV (Leiden, The Netherlands). A paired-end DNA library with a mean gap length size between 230 and 360 bp was sequenced, with average reads of 100 bp, on an Illumina HiSeq2500 apparatus (Illumina Inc., San Diego, CA, USA). Quality trimmed reads were obtained by quality trimming in CLC Genomics Workbench v6.5.1 (CLC Inc., Aarhus, Denmark) based on a quality score limit of 0.05 and a maximum number of ambiguous nucleotides of 2. Quality trimming was based on the Phred quality scores (Q) from the fastq read files as follows. The Q value was used to calculate an error probability (p_error_ = 10^Q/-10^). Next, for every base a new value was calculated (limit – p_error_). This value was negative for low-quality bases. For every base, the CLC workbench calculated the running sum of this value. If the sum dropped below zero, it was set to zero. The part of the sequence not trimmed was the region ending at the highest value of the running sum and started at the last zero value before this highest score. Everything in front and behind this region was trimmed. With the ambiguous trim set to 2, the algorithm found the maximum length region containing 2 or fewer ambiguities and then trimmed away the ends not included in this region. Trimmed reads shorter than 15 bp were discarded. After trimming, an initial *de novo* assembly was performed in CLC Genomics Workbench v6.5.1, using the quality-trimmed paired reads. All contigs shorter than 500 bp were discarded. Contigs were ordered automatically with MAUVE [38] by comparing the draft genome sequence of *P. damnosus* LMG 28219 with the complete chromosomal DNA of *P. claussenii* ATCC BAA-344^T^. Contigs that did not show similarities towards the *P. claussenii* ATCC BAA-344^T^ chromosomal DNA were blasted against a plasmid and phage database, using the PATRIC (Pathosystems Resource Integration Center) [39] and PHAST (PHAge Search Tool) [40] websites, respectively. In addition, the average GC content of the contigs was calculated using an in house developed Python script.

### Genome annotation

Functional annotation and metabolic reconstruction were performed with (i) the Rapid Annotation Subsystem Technology (RAST) server [41], using GLIMMER [42] for gene calling and allowing frameshift corrections, backfilling of gaps, and automatic fixing of errors; (ii) the Integrated Microbial Genomics Expert review (IMG-ER) annotation pipeline, using GenePRIMP for gene prediction [43]; and (iii) the National Center for Biotechnology Information’s Prokaryotic Genome Automatic Annotation Pipeline (PGAAP), which uses GeneMark and GeneMark.HMM for gene calling [44]. The automated gene prediction and annotation of PGAAP was followed by manual curation of the CDSs of interest, using the results of the IMG-ER and RAST server, BLASTp [45], UniProt (http://www.uniprot.org/), and InterProScan [36].

### Comparative genomics

Comparative genomics of the draft genome sequence of *P. damnosus* LMG 28219 was performed using the available complete genome sequences of *P. claussenii* ATCC BAA-344^T^ and *P. pentosaceus* ATCC 25745. The average nucleotide identities (ANIs) between the draft genome (*P. damnosus* LMG 28219) and both reference genomes were calculated using the *in silico* DNA-DNA hybridization method implemented in the Jspecies software [46], using BLAST, as proposed by Goris and colleagues [47]. The OrthoMCL tool was used with default settings for ortholog finding in the genomes of *P. damnosus* LMG 28219, *P. claussenii* ATCC BAA-344^T^, and *P. pentosaceus* ATCC 25745 (http://orthomcl.org/). The Kyoto encyclopedia of genes and genomes (KEGG) database was used for the reconstruction of the folate biosynthesis metabolic map (http://www.genome.jp/kegg/).

### Availability of supporting data

The raw sequence data received from BaseClear BV were deposited at the Short Read Archive (SRA) of GenBank (accession number SRP035530). This Whole Genome Shotgun project was deposited at DDBJ/EMBL/GenBank under the accession number JANK00000000 after automatic annotation by PGAAP and manual curation of the genes of interest.

## Additional files

Additional file 1:
**Overview of the GC content and the length of the contigs of the draft genome of** ***Pediococcus damnosus*** **LMG 28219.** no.: number, bp: base pair. Additional file 2:
**Shared and unique orthologous proteins of** ***Pediococcus damnosus*** **LMG 28219,** ***P. claussenii*** **ATCC BAA-344** ^**T**^ **, and** ***P. pentosaceus*** **ATCC 25745.** clau: *P. claussenii* ATCC BAA-344^T^, ldam: *P. damnosus* LMG 28219, pent: *P. pentosaceus* ATCC 25745 Both the locus tag, protein ID and annotation of the CDSs are listed.
